# Mid-Burdigalian Paratethyan alkenone record reveals link between orbital forcing, Antarctic ice-sheet dynamics and European climate at the verge to Miocene Climate Optimum

**DOI:** 10.1016/j.gloplacha.2014.10.011

**Published:** 2014-12

**Authors:** Patrick Grunert, Alexandrina Tzanova, Mathias Harzhauser, Werner E. Piller

**Affiliations:** a Institute for Earth Sciences, University of Graz, NAWI Graz, Heinrichstraße 26, 8010 Graz, Austria; b Department of Geological Sciences, Brown University, 324 Brook Street, Box 1846, Providence, RI 02912, USA; c Geological–Paleontological Department, Natural History Museum Vienna, Burgring 7, 1030 Vienna, Austria

**Keywords:** Paratethys, Burdigalian, Early Ottnangian Cooling, alkenones, palaeoclimate, orbital forcing

## Abstract

The Early Ottnangian Cooling (EOC), a distinct cold-spell in European climate at ~ 18 Ma preceding the Miocene Climate Optimum, is frequently reported in Paratethys records; however, the duration, magnitude, and underlying causes are poorly understood. A new palaeoclimatic data-set provides unexpected insights into this event.

U^K'^_37_-based sea-surface temperatures > 24 °C between ~ 18.1 and 17.7 Myrs substantially exceed existing estimates, and indicate a significantly warmer European climate than previously assumed for this usually poorly recovered time interval. The EOC is expressed as an average drop of 2–3 °C in Paratethyan water temperatures between ~ 18.1 and 17.8 Myrs with two distinct cold snaps at ~ 17.86 Ma and ~ 17.81 Ma. The short duration of the EOC excludes Tethyan Seaway closure as its underlying cause, although the enhanced palaeoclimatic sensitivity of the Paratethys due to this palaeogeographic configuration potentially contributed to the magnitude of SST deterioration during the EOC. The revealed palaeoclimatic pattern shows a strong correlation with isotope event Mi-1b in deep-sea δ^18^O records, and we propose a tight palaeoclimatic link between the Southern Ocean and the Paratethys/Mediterranean realm as an alternative hypothesis. The interplay of modulations in the long-term (~ 400 kyrs) and short-term (~ 100 kyrs) eccentricity cycles most likely acted as pacemaker of this palaeoclimatic interaction.

## Highlights

• New insights into Early Ottnangian Cooling (EOC) from U^K'^_37_ data (18.1–17.7 Myrs)
• Tropical sea surface temperatures > 24 °C during the EOC exceed previous estimates.
• Two cold snaps at ~ 17.86 Ma and ~ 17.81 Ma punctuate the EOC record.
• Results indicate palaeoclimatic link between Mi-1b glaciation and EOC.
• Paced by long-term (~ 400 kyrs) and short-term (~ 100 kyrs) eccentricity forcing

## 1 Introduction

The palaeoclimatic records of the Central Paratethys Sea indicate a distinct middle Burdigalian episode of climate deterioration (Early Ottnangian Cooling; EOC) (Hochuli, 1978) that immediately precedes the Miocene Climate Optimum (Böhme, 2003; Zachos et al., 2008). Although recognized in terrestrial (Hochuli, 1978; Rögl et al., 1979; Nagy, 2005) and marine (Nebelsick, 1989, 1992; Jenke, 1993; Chira, 2004; Janz and Vennemann, 2005; Kroh, 2007; Kocsis et al., 2009) proxy data from different basins of the Central Paratethys, its timing, magnitude, and underlying causes have remained largely speculative. Some authors have attributed the EOC to the closure of the Tethyan Seaway (Karami et al., 2009, 2011), and the cessation of circum-equatorial circulation (Nebelsick, 1989; Jenke, 1993). However, due to insufficient stratigraphic and palaeoclimatic constraints, evidence for this hypothesis has remained ambiguous (cf. Karami et al., 2011).

We present a new palaeoclimatic proxy record from marine deposits of the North Alpine Foreland Basin (NAFB; in many studies referred to as Molasse Basin), a prime study area for the EOC due to exceptionally high sedimentation rates. The data-set provides new insights into European climate between ~ 18.1 and ~ 17.7 Myrs, an interval otherwise poorly recovered in the sedimentary records of continental Europe and the Mediterranean Sea. The alkenone-based U^K'^_37_ index allowed for the calculation of Paratethyan sea-surface temperature (SST), a quantitative evaluation of the magnitude, duration, and internal variability of the EOC, and a new assessment of its underlying causes.

## 2 Regional setting

The middle Burdigalian sediments studied in here are part of the Cenozoic infill (“Molasse”) of the NAFB, which extends from Savoy (France) in the west to Lower Austria in the east (Bachmann et al., 1987; Malzer et al., 1993; Wagner, 1998; Kuhlemann and Kempf, 2002). The tectonic, palaeogeographic and depositional history of the NAFB is strongly related to Alpine orogeny and the disintegration of the Tethys Sea during the early Cenozoic (Bachmann et al., 1987; Malzer et al., 1993; Berger, 1996; Rögl, 1998; Wagner, 1998; Kuhlemann and Kempf, 2002; Harzhauser and Piller, 2007). Around the Eocene/Oligocene boundary the rising Alpine chains formed a biogeographic barrier and divided the Tethys into the northern Paratethys and the southern Mediterranean (Rögl, 1998; Harzhauser and Piller, 2007). Subsequently, the NAFB acted as one of the main depositional basins of the Central Paratethys until the middle Burdigalian when its infill and a major palaeogeographic reorganization culminated in the eastward regression of the sea (Rögl, 1998; Wagner, 1998). The herein studied deposits represent this terminal phase of marine sedimentation in the NAFB and belong to the regional substages lower and middle Ottnangian of the Central Paratethys (~ 18.1–17.5 Myrs; Rögl, 1998; Piller et al., 2007; Grunert et al., 2010, 2012; Pippèrr, 2011; see next chapter for a review of the Ottnangian substages).

## 3 Material and methods

### 3.1 Studied sites and sample material

**Fig. 1:**
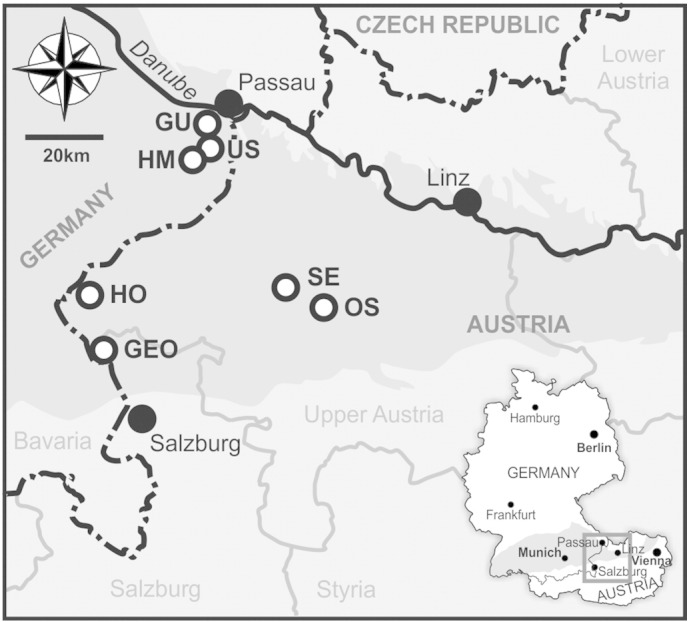
Localities in the NAFB evaluated for U^K'^_37_. GEO = St. Georgen 1, GU = Gurlarn, HM = Höhenmühle, HO = Hochburg 1, OS = Ottnang-Schanze, SE = Straß-Eberschwang, US = Untersimbach. Distribution of Cenozoic sediments in the NAFB study area is indicated in dark grey colour.

22 samples from seven middle Burdigalian (lower to middle Ottnangian) localities in the central part of the NAFB in north-eastern Austria and south-eastern Germany have been used for SST reconstruction from alkenones in the present study (Table 1; Fig. 1). The selected sites consist of borehole and outcrop sections for which extensive information on stratigraphic constraints is available from previous studies (see below).

#### 3.1.1 Hochburg 1

This borehole, drilled by Rohöl-Aufsuchungs AG (RAG) in 1983, is located in western Upper Austria (N 48°12′22″; E 14°13′27″). Extensive stratigraphic information (lithostratigraphy, biostratigraphy, sequence stratigraphy, well-log data, seismic images) on the lower and middle Burdigalian deposits of this site has been published by Grunert (2009) and Grunert et al. (2013). Lower Ottnangian sediments reach a thickness of 550 m and consist of sandy–silty marls with intercalations of sand layers deposited in an outer neritic environment (Grunert, 2009; Grunert et al., 2013). Three samples have been taken from the upper portion of this sequence at 560 m, 490 m, and 450 m. The middle Ottnangian deposits are 319 m-thick and show a more diverse lithology reflecting the shallowing environment as a consequence of the retreating sea. Silty–sandy marls continue in the lower, 75 m-thick part of the middle Ottnangian (Ried Fm.) whereas thick intercalations of sands and gravel characterize the middle and upper parts (Grunert, 2009). One sample has been taken from the middle Ottnangian Ried Fm. at 400 m close to the lower/middle Ottnangian boundary.

#### 3.1.2 St. Georgen 1

Located ~ 20 km south of Hochburg 1 (N 47°59′22″; E 12°53′35″), this borehole was drilled by RAG in 1990. Stratigraphic information (lithostratigraphy, biostratigraphy, well-log data, seismic images) for this site has been compiled from internal reports provided by RAG as well as site-to-site correlation of seismic marker horizons between St. Georgen 1 and Hochburg 1. For the most part, the 578 m-thick lower Ottnangian succession consists of silty–sandy marls; only in its lower part, a 70 m-thick package of gravel is intercalated with the marls and has been avoided during sampling. The position along the southern margin of the NAFB and the microfossil content indicate deposition in a neritic environment shallower than at Hochburg 1. One sample has been taken from the base of the Ottnangian at 650 m, and one sample from the middle part at 320 m.

#### 3.1.3 Untersimbach

This ~ 4 m-thick outcrop, located 11 km SW of Passau (N 48°29′17″; E 13°21′49″) exposes parts of the Untersimbach Beds that represent the lowermost Ottnangian in the German study area (Wenger, 1987; Doppler et al., 2005; Pippèrr, 2011). Foraminiferal content suggests deposition of the finely laminated alternations of brownish marls and sands in a middle neritic setting (Pippèrr, 2011). Four samples (US 1, 3, 4, 5) have been taken in regular intervals from this outcrop.

#### 3.1.4 Ottnang-Schanze

This ~ 10 m-thick lower Ottnangian section (N 48°06′07″; E 13°40′04″) represents the (facio)stratotype of the Ottnangian stage and has recently been re-evaluated for stratigraphy (Rögl et al., 1973; Grunert et al., 2010). The characteristic sandy–silty marls correspond to the lower part of the Ottnang Fm. and show a coarsening upward trend (Rupp et al., 2008; Grunert et al., 2010). The coarsening deposits represent the shallowing outer-middle neritic environment in the central part of the basin (Grunert et al., 2012). Magneto- and biostratigraphy suggest an age of deposition between 18.06 Ma and 17.95 Ma (Grunert et al., 2010; also see Results section). Six samples (OS 1, 5, 10, 13, 17, 22; Grunert et al., 2010) from different intervals of this site have been included herein.

#### 3.1.5 Höhenmühle

This outcrop ~ 15 km SW of Passau (N 48°28′36″; E 13°16′13″) exposes the lower/middle Ottnangian boundary (Wenger, 1987; Rupp et al., 2008; Pippèrr, 2011). Sediments show intercalations of marls and sands deposited in a shallow marine environment (Pippèrr, 2011). Three samples have been taken within a few metres of the section from the uppermost Neuhofen Beds (lower Ottnangian; HM 2) and the boundary interval (HM 3, HM 4; transition from lower Ottnangian Neuhofen Beds to middle Ottnangian Glaukonitsande & Blättermergel; Pippèrr, 2011).

#### 3.1.6 Gurlarn

Similar to Höhenmühle, this section ~ 9 km SW of Passau (N 48°31′32″; E 13°20′11″) comprises the uppermost Neuhofen Beds and the lower part of the middle Ottnangian Glaukonitsande & Blättermergel, thus spanning the lower/middle Ottnangian boundary (Frieling et al., 2009). The often-laminated silty–sandy marls with intercalations of sand have been deposited in an inner neritic environment (Frieling et al., 2009). Two samples from the boundary interval (GU 1) and the middle Ottnangian (GU 2) have been included.

#### 3.1.7 Straß-Eberschwang

~ 13 m of the middle Ottnangian Ried Fm. is exposed at this site consisting of sandy silts deposited during lower nanoplankton zone NN4 (Rupp et al., 2008). The palaeoenvironment is considered a shallow marine (Jiménez-Moreno et al., 2006; Rupp et al., 2008). One sample (SE 7) from the lower part of the section has been taken.

### 3.2 Ottnangian substage boundaries and age model

**Fig. 2:**
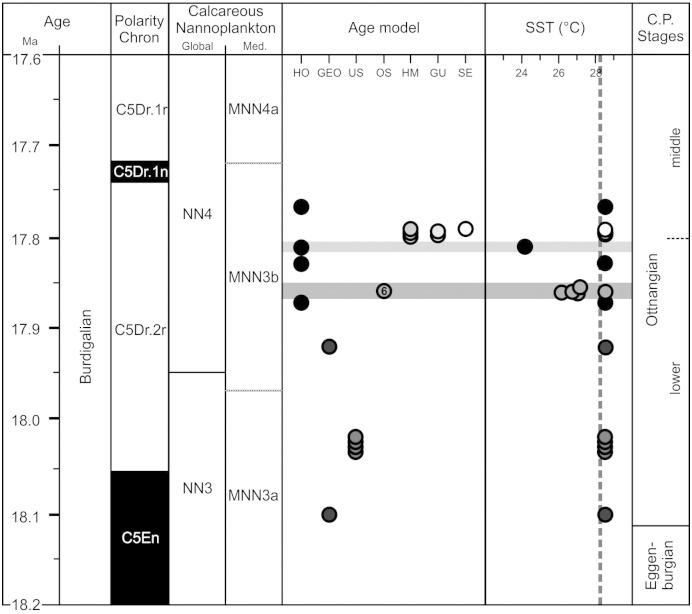
Middle Burdigalian stratigraphy, age model, and U^K'^_37_-based SSTs. Correlation of the regional (sub)stages is based on Piller et al. (2007), Pippèrr et al. (2007), and Hilgen et al. (2012). For details on the age model and stratigraphic error margins see text and Tables 1 and 2. Solid dark grey lines highlight the two identified EOC cold snaps; stippled line marks the upper limit of 28.2 °C for SST reconstruction from alkenones. The number “6” in OS column refers to the number of samples in this very narrow time interval represented by Ottnang-Schanze. See Fig. 1 for abbreviations of individual localities. C.P. = Central Paratethys.

A major shortcoming of previous EOC studies is the lack of a reliable stratigraphic framework. For the present study, a new age model has been developed based on the critical review and integration of available stratigraphic data-sets. Geochronological constraints for three marker horizons (base lower Ottnangian, base middle Ottnangian, end of marine sedimentation in study area) have been defined to represent distinct lithostratigraphic boundaries. They are easy to identify in seismic images (Zweigel, 1998; Grunert et al., 2013; Table 2) and serve as the basis of the applied age model. Individual ages have then been determined for each sample of the boreholes Hochburg 1 and St. Georgen 1 based on their position relative to the marker horizons. Finally, the outcrop samples have been arranged relative to the borehole samples based on constraints from litho-, bio-, and magnetostratigraphy as well as seismic images (Table 1; Fig. 2).

#### 3.2.1 Base lower Ottnangian

Based on the transgressive character of basal Ottnangian deposits throughout the Central Paratethys basins, Piller et al. (2007) and Hilgen et al. (2012) argue for a correlation of the lower Ottnangian boundary with global 3rd-order sequence boundary Bur 3 at 18.12 Ma. In the study area, recent investigations on the transgressive lower Ottnangian sediments have revealed strong evidence that supports this correlation (Hinsch, 2008; Pippèrr, 2011; Grunert et al., 2013). Given the evidence we consider the datum of 18.12 Ma as most plausible; however, it represents only the youngest of several available estimates. Older ages range between 18.2 and 18.5 Myrs (e.g., Abdul Aziz et al., 2009; Reichenbacher et al., 2013). However, an age older than 18.28 Ma for the base of the Ottnangian is unlikely as no Ottnangian deposits from nanoplankton zone NN2 have been recovered so far (Rögl et al., 1979; Rögl, 1998; Grunert et al., 2010, 2013; Hilgen et al., 2012).

#### 3.2.2 Base middle Ottnangian

Fully marine conditions prevailed in the study area for most of the middle Ottnangian (Kuhlemann and Kempf, 2002; Rupp et al., 2008; Pippèrr, 2011). An absolute age of 17.8 ± 0.3 Ma for the base of the middle Ottnangian has been suggested by Pippèrr et al. (2007) based on Sr isotope data from the southern margin of the NAFB. The error margin of this datum is narrowed by bio- and magnetostratigraphic constraints: middle Ottnangian deposits range within the lowermost part of nanoplankton zone NN4, and consequently have to be younger than 17.95 Ma (Hochuli, 1978; Rupp et al., 2008; Hilgen et al., 2012). The upper error margin can be constrained by magnetostratigraphy to an interval between 17.74 Ma (base C5Dr.1n) and 17.533 (top C5Dr.1r; Reichenbacher et al., 2013; see next paragraph).

#### 3.2.3 End of marine sedimentation in the NAFB

The eastward retreat of the Paratethys from the NAFB resulted in a patchy and diachronous distribution of marine, brackish and fluvial middle Ottnangian deposits in the NAFB, which complicates the dating of the end of marine sedimentation in the study area. Magneto- and biostratigraphic data from the NAFB west of Munich (where the retreat occurred slightly earlier than in the study area) indicate deposition of marine deposits well within chron 5Dr.1r, and speculative dates between 17.6 and 17.5 Ma have been suggested (Böhme, 2003; Böhme et al., 2007; Reichenbacher et al., 2013). However, erosion and debatable biostratigraphy make any exact estimate uncertain. For these reasons in our age model we use the top of chron 5Dr.1r at 17.533 Ma as a conservative estimate of the youngest age for the end of marine sedimentation. Based on an extrapolation of lower Ottnangian sedimentation rates into the middle Ottnangian we also consider the base of chron 5Dr.1n at 17.74 Ma as a possible constraint as in this study the middle Ottnangian is represented only with its basal part (Ried Fm.).

### 3.3 Sea-surface temperatures

SST estimates are based on the reconstruction of the U^K′^_37_ index from lithified marine sequences (Cleaveland and Herbert, 2009; Beltran et al., 2011). Samples were homogenized using a mortar and pestle. Lipid extractions were done on a Dionex 200 Accelerated Solvent Extractor. Total lipid extract was obtained from ~ 10 g of sediment using 9:1 DCM:MeOH. Samples underwent silica gel separation prior to gas chromatographic analysis to ensure reliable alkenone quantification and minimize system drift. Alkenone concentrations were quantified on an Agilent 6890N GC-FID and SST calculated following the Prahl and Wakeham (1987) calibration of the U^K^'_37_ index. Drift and error were monitored by including sample replicates and a lab standard every 12 samples. The analytical error of the laboratory as determined by replicates is 0.2 °C.

In previous EOC studies, climatic conditions have been described in qualitative terms, e.g. *temperate* and *subtropical*. We assign the following SST ranges to the respective qualitative terms (Karami et al., 2011): temperate = 15–17 °C; warm temperate = 17–21 °C; subtropical = 21–25 °C; tropical = > 25 °C.

## 4 Results

### 4.1 Age model

Table 1 indicates three ages for each sample from the boreholes Hochburg 1 and St. Georgen 1 based on the available stratigraphic constraints: a most likely age as well as maximum (oldest) and minimum (youngest) ages, the later serving as upper and lower error margin for each estimate. The calculations suggest a time frame between 18.10 and 17.77 Myrs for the studied samples based on the ages considered to be most likely. The average error envelope is 0.31 Ma with a minimum stratigraphic range between 18.09 and 17.84 Myrs and a maximum stratigraphic range between 18.26 and 17.66 Myrs.

The calculated most likely ages for each section are generally in good agreement with the available stratigraphic constraints. Only the depositional age for the OS samples (17.85–17.86 Myrs) reveals results contradictory to the previous study of Grunert et al. (2010) who suggested a depositional age of 18.06–17.95 Myrs. While the herein calculated younger age agrees well with the correlation of Ottnang-Schanze to polarity chron C5Dr.2r, the suggested time interval falls within the lowermost nanoplankton zone NN4 in contrast to upper NN3 as suggested by Grunert et al. (2010). However, Grunert et al. (2010) point out that the index taxa *Sphenolithus belemnos* and *Sphenolithus heteromorphus* are rare and that the assemblages show clear affinities with the Mediterranean zone MNN3b. This zone ranges well into zone NN4 (Fig. 2; Fornaciari and Rio, 1996), and we thus argue that our most likely age is valid and the biostratigraphic interpretation of Grunert et al. (2010) should be re-considered in that respect (there is no contradiction with dinoflagellate cysts and benthic foraminifers).

### 4.2 Alkenone record and SST reconstruction

Throughout the studied interval, SSTs exceed 28.2 °C, the lower bound on annual temperatures; once the U^K'^_37_ index reaches a value of 1, the magnitude of warmth cannot be resolved (Herbert, 2003). Deviations from the high SSTs occur as two cold snaps, i.e. short periods of extreme climatic deterioration (Table 2; Fig. 2): (1) SSTs from 26.2 °C to 28.2 °C occur between ~ 17.85 Ma and ~ 17.86 Ma (max. range: 18.02–17.59 Myrs); (2) the lowest SST of 24.1 °C for all samples has been calculated for sample HO 450 (~ 17.81 Ma) (max. range: 17.96–17.55 Myrs). The SST value of the later sample has to be interpreted with caution as alkenone concentrations in the sample are near the detection limit. However, there is isotopic evidence of a considerable deterioration of water temperatures at this time (Janz and Vennemann, 2005; see Discussion section), and the sample has thus been included in the analysis.

## 5 Discussion

### 5.1 Tropical conditions during the middle Burdigalian?

In earlier studies, the cooling of Paratethyan water temperatures has been described as a temporary deterioration from subtropical to warm-temperate conditions, manifested in the development of lower Ottnangian bryozoan carbonates (Nebelsick, 1989, 1992). The herein calculated SSTs > 24 °C even for the coolest samples indicate substantially warmer conditions for the middle Burdigalian. Although the results contradict previous interpretations, the persisting tropical conditions are in agreement with other proxy records. Strong support arises from the frequent abundance of the dinoflagellate cyst *Polysphaeridium zoharyi* in shallow marine deposits of the NAFB including the site Ottnang-Schanze (Jiménez-Moreno et al., 2006; Grunert et al., 2012). This euryhaline species thrives in coastal areas of subtropical and tropical oceans, and is widely used as a palaeoclimatic indicator (Pross and Brinkhuis, 2005). Optimum growth conditions of this species are related to summer SSTs > 28 °C (Marret and Zonneveld, 2003), and high abundances during the EOC support the inferred tropical SSTs (Grunert et al., 2012).

While there is sound evidence for tropical SSTs during the EOC, the examination of proxy records previously related to temperate conditions reveals eurythermy of the involved palaeoclimatic indicators as a likely bias. Bryozoan carbonates in lower Ottnangian shallow water deposits (Nebelsick, 1989, 1992; Pippèrr, 2011) have been regarded as the prime indicator of temperate conditions at the time of EOC. However, recent studies severely question the viability of bryozoan carbonates as palaeoclimatic indicators, and report them in subtropical and tropical areas as well depending on local/regional environmental conditions, e.g., raised nutrient content (Pomar et al., 2004). Increased terrigenous runoff and high nutrient availability can also be cited for the lower Ottnangian carbonates precluding coral reef growth. However, z-corals, indicative for tropical/subtropical climate, are well reported from these deposits (Steininger and Seneš, 1971; Perrin and Bosellini, 2012). The same applies for foraminiferal assemblages associated with the bryozoan carbonates and related to temperate conditions based on the larger foraminifers *Amphistegina lessonii* and *Sphaerogypsina globulosa* (Jenke, 1993). However, extant *A. lessonii* specimens require a winter isotherm of 14 °C, and are restricted to the southern coasts of the Mediterranean Sea where they thrive under tropical summer SSTs (Langer and Hottinger, 2000).

### 5.2 Duration, internal variability, and magnitude of the EOC

Previous studies indicate that the EOC lasted from the base of the Ottnangian (Sandmergelhorizont; Kocsis et al., 2009) to the lower/middle Ottnangian boundary (upper Neuhofen Beds; Janz and Vennemann, 2005). The duration of the EOC can thus be reasonably constrained to ~ 300–400 kyrs beginning at ~ 18.1 Ma.

**Fig. 3:**
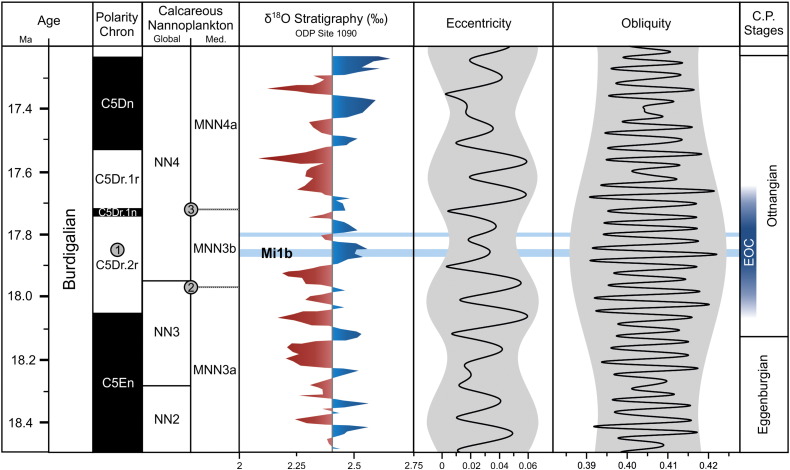
Correlation of the EOC with isotope event Mi-1b (ODP Site 1090) and orbital forcing. Numbers in circles indicate magnetostratigraphic (Billups et al., 2002) and biostratigraphic (Raffi et al., 2006) constraints: (1) position of Mi-1b relative to the base of chron C5Dr; (2) highest consistent occurrence of *Sphenolithus belemnos*, ~ 17.97 Ma; (3) lowest consistent occurrence of *Sphenolithus heteromorphus* ~ 17.72 Ma. Positive and negative deviations from mean δ^18^O in the studied interval are indicated in blue and red, respectively. Black lines show short-term variations in eccentricity (~ 100 kyrs) and obliquity (~ 41 kyrs), and long-term modulations (~ 400 kyrs and ~ 1200 kyrs, respectively) are highlighted in grey colour (Laskar et al., 2004). EOC cold snaps identified from the alkenone record are indicated by light blue bars, and the duration of the EOC is indicated together with the Central Paratethys (C.P.) stages.

The SST record allows for the first time an evaluation of palaeoclimatic variability within the EOC interval, expressed as cold snaps. The first drop in SST between ~ 17.85 Ma and ~ 17.86 Ma is well recorded at Ottnang-Schanze (Fig. 3). Given its stratigraphic position it most likely corresponds to the positive δ^18^O peak reported previously from the NAFB (Neuhofen Beds; Janz and Vennemann, 2005). As pointed out before, the second event at ~ 17.81 Ma has to be treated with caution. However, its validity is supported by a large positive shift in δ^18^O close to the lower/middle Ottnangian boundary (uppermost Neuhofen Beds; Janz and Vennemann, 2005).

The average δ^18^O increase of 0.5–0.75‰ in the EOC interval suggests an average decrease of 2–3 °C throughout the water column (Janz and Vennemann, 2005; Kocsis et al., 2009). The alkenone data indicate a further decrease of SSTs during the two cold snaps of at least 2 °C, which is in agreement with a decrease of 4–5 °C during these extreme events compared to the late Eggenburgian and middle Ottnangian (Janz and Vennemann, 2005; Kocsis et al., 2009).

### 5.3 Underlying causes of the EOC

Our interpretation of the palaeotemperature data questions the depiction of EOC as a phenomenon induced by a significant change of oceanic heat transport resulting from Tethyan Seaway closure and cessation of circum-equatorial circulation (Nebelsick, 1989; Karami et al., 2009, 2011). This hypothesis has been supported by palaeoceanographic models that estimate a progressive decrease of ~ 4 °C in Mediterranean and Paratethys water temperatures as a result of the disconnection from the Indian Ocean (Karami et al., 2009, 2011). In light of the new information, this hypothesis does not account for the EOC for two reasons. First, Tethyan Seaway closure resulted from a long and complex process over 1–2 Myrs, and restrictions of the strait already occurred in the late Aquitanian and early Burdigalian, thus long before the EOC onset (Harzhauser et al., 2007; Reuter et al., 2009). Second, the hypothesis fails to explain the short duration of the EOC as the Tethyan Seaway remained closed for another two million years (Harzhauser et al., 2007).

Previous studies indicate that Paratethyan water temperatures and European climate during the Neogene largely follow Antarctic ice-sheet dynamics, which in turn were preconditioned by orbital forcing (Bicchi et al., 2003; Janz and Vennemann, 2005; Van Dam et al., 2006; Kocsis et al., 2009; Utescher et al., 2012). Phases of increased Antarctic ice-volume are recorded as positive excursions in deep-sea δ^18^O records (Mi-events; Miller et al., 1991), and many of these events have been recognized in the Mediterranean and Paratethys seas (e.g., Turco et al., 2001; Bicchi et al., 2003; Janz and Vennemann, 2005; Kocsis et al., 2009; De Leeuw et al., 2010; Foresi et al., 2013). None of the later studies discusses the EOC specifically but relate a synchronous positive δ^18^O shift in the Paratethys and an increase in cold-water species of planktonic foraminifera in the Mediterranean to isotopic event Mi-1b (Miller et al., 1991; Janz and Vennemann, 2005; Kocsis et al., 2009; Foresi et al., 2013, 2014). One of the best archives of Early Miocene ice-volume changes is provided by the well-calibrated δ^18^O record of OPD Site 1090 (Billups et al., 2002; Raffi et al., 2006). There, magneto- and biostratigraphy constrain the Mi-1b event to polarity chron C5Dr.2r and the transition between calcareous nanoplankton zones NN3 and NN4 (Fig. 3; Billups et al., 2002; Raffi et al., 2006). The generally increased δ^18^O values in this interval are punctuated by a series of positive peaks, of which the first ranges between ~ 17.83 Ma and ~ 17.87 Ma and is defined as Mi-1b (Fig. 3; Billups et al., 2002). A comparison with the alkenone data shows that the EOC matches the δ^18^O record very well on the level of variability: the cold snaps at ~ 17.85–17.86 and ~ 17.81 Ma resemble Mi-1b and the subsequent peak (Fig. 3). This remarkable resemblance strongly implies a palaeoclimatic link between Antarctic ice-sheet dynamics and the Paratethys/Mediterranean realm during the EOC.

Orbital forcing plays a pivotal role in driving coherent changes in Antarctic ice-volume and the carbon cycle, and thus global climate (Zachos et al., 2001a; De Boer et al., 2013). Specifically the interplay of modulations in the long-term (~ 400 kyrs) and short-term (~ 100 kyrs) cycles of eccentricity has been identified as the primary pacemaker of glaciation events since the Oligocene (Zachos et al., 2001a; Pälike et al., 2006; Holbourn et al., 2007, 2013; Liebrand et al., 2011; De Boer et al., 2013). Only with extreme glaciation events (e.g., Oi-1, Mi-1, Mi-3b) the concurrence of eccentricity minima and low-amplitude variations (nodes) in the long-term (~ 1.2 Myrs) obliquity cycle have to be taken into account as an additional component (Lourens and Hilgen, 1997; Zachos et al., 2001b; Pälike et al., 2006; Van Dam et al., 2006; Holbourn et al., 2007). Mi-1b and the coolest phase of the EOC follow this general pattern as they occur during a minimum node in the long-term (~ 400 kyrs) eccentricity cycle and low-amplitude short-term (~ 100 kyrs) eccentricity cycles, an orbital configuration that has been suggested as the primary trigger of Antarctic ice-sheet growth during the Early Miocene (Fig. 3; Laskar et al., 2004; Pälike et al., 2006; Liebrand et al., 2011). It is noteworthy that the EOC onset predates Mi-1b by 100–200 kyrs and coincides with the beginning of a declining 400 kyrs-eccentricity cycle (Fig. 3). The EOC onset might thus reflect the initiation of Antarctic ice-sheet growth.

In stark contrast to the prominent Mi-3b event that marks the transition into the “ice-house” phase of Miocene climate (Holbourn et al., 2007) and which has similarly been linked to a severe deterioration of Paratethys SSTs at ~ 13.81 Ma (Bicchi et al., 2003; De Leeuw et al., 2010), Mi-1b and the EOC fall within a maximum of the long-term (~ 1.2 Myrs) obliquity cycle (Fig. 3). Obliquity largely determines seasonal contrast at high-latitudes, and a maximum in its amplitude variation might have slowed Antarctic ice-sheet growth to a certain amount and contributed to the small-scale nature of Mi-1b compared to Mi-3b. While the link between eccentricity forcing, the Mi-1b glaciation and the EOC seems well supported by the new data, the magnitude of the EOC requires additional consideration in the light of the potentially tempering effect of obliquity on Antarctica. Although its long duration excludes Tethyan Seaway closure as an immediate trigger, the disconnection from the Indian Ocean might have indirectly contributed to the strength with which the EOC is expressed in the Paratethys records. Modelling studies by Karami et al. (2011) indicate that the restricted exchange with the open ocean resulted in an increased sensitivity of the Paratethys to climate change and atmospheric forcing.

In the light of the new evidence, we propose positive feedback mechanisms between orbital forcing, Antarctic ice-sheet dynamics and European climate, possibly enhanced by a heightened palaeoclimatic sensitivity of the Paratethys and Mediterranean seas, as an alternative hypothesis that accounts for the shortcomings of the seaway hypothesis and reasonably explains the observed palaeoclimatic pattern during the EOC.

## 6 Conclusions

A new palaeoclimatic data-set from middle Burdigalian (18.1–17.7 Myrs) deposits of the Central Paratethys reveals important information on circum-Mediterranean climate on the verge to the Miocene Climate Optimum. The record spans an otherwise poorly recovered time interval, and includes the distinctive Early Ottnangian Cooling, a short period of climate deterioration that has been poorly constrained and understood so far.

Alkenone-based SSTs > 24 °C, prevailing throughout the investigated time period, substantially exceed existing estimates, and indicate a significantly warmer climate than previously assumed for the area. Tropical conditions are also supported by the frequent occurrence of the dinoflagellate cyst *P. zoharyi* in lower and middle Ottnangian shallow water deposits. The EOC corresponds to an average decrease of 2–3 °C in Paratethyan water temperatures between ~ 18.1 and 17.8 Myrs with two distinct periods of extreme cooling at ~ 17.86 Ma and ~ 17.81 Ma identified from the new data. This palaeoclimatic pattern shows a strong correlation to isotope event Mi-1b as identified in deep-sea δ^18^O records of the Southern Ocean and occurs during a minimum node in the long-term (~ 400 kyrs) and low-amplitude short-term (~ 100 kyrs) eccentricity cycles, an orbital configuration highly favourable for Antarctic ice-sheet growth in the Early Miocene. The tight synchronicity between the Southern Ocean and the Paratethys/Mediterranean realm suggests positive feedback mechanisms between orbital forcing, Antarctic ice-sheet expansion and global climate, possibly enhanced by an increased palaeoclimatic sensitivity of the Paratethys due to Tethyan Seaway closure, as a plausible hypothesis to explain the EOC.

## Figures and Tables

**Table 1:**
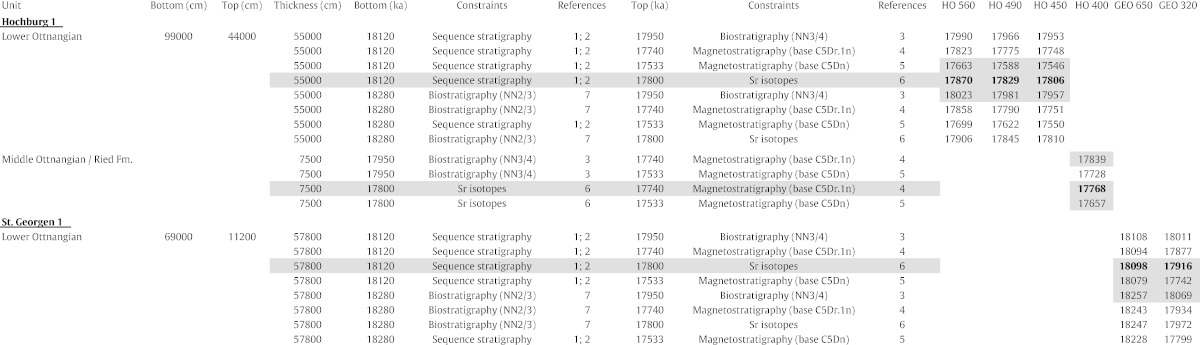
Stratigraphic constraints for the herein applied age model and calculated ages for the samples from boreholes Hochburg 1 and St. Georgen 1. Most likely (bold numbers), maximum and minimum ages are highlighted in grey for each sample. See text for details and discussion of the age model. Reference numbers refer to: (1) Piller et al. (2007); (2) Grunert et al. (2013); (3) Grunert et al. (2010); (4) Abdul Aziz et al. (2009); (5) Reichenbacher et al. (2013); (6) Pippèrr et al. (2007); (7) Rögl et al. (1979).

**Table 2:**
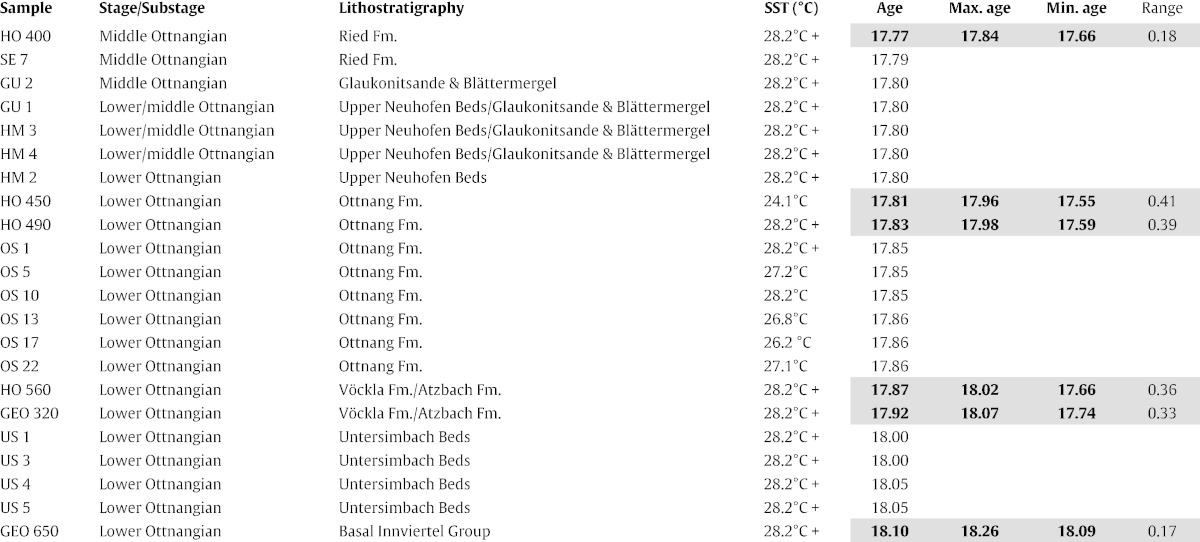
Calculated SSTs and age estimates for the studied samples. For details on the age model see text and Table 1.
